# Validation of the Arabic Version of Feeding Handicap Index for Children with Developmental Disabilities (A-FHI-C)

**DOI:** 10.1007/s10803-024-06289-3

**Published:** 2024-04-02

**Authors:** Nesreen Fathi Mahmoud, Zeinab Mohammed, Hassnaa Othman Mohammed, Alshimaa Mohsen Mohamed Lotfy

**Affiliations:** 1 Phoniatrics Unit-Department of ENT, Faculty of Medicine, Beni-Suef University, Beni-Suef, Egypt; 2 Department of Public Health and Community Medicine, Beni-Suef University, Beni-Suef, Egypt; 3 Medical Studies Department for children, Faculty of Postgraduate Childhood Studies, Ain Shams University, Cairo, Egypt; 4 Department of Public Health and Community Medicine, Faculty of Medicine, Beni-Suef University, Beni-Suef, Egypt

**Keywords:** Feeding handicap index for children, Arabic version, Autism spectrum disorder, Cerebral palsy, Intellectual disability, Developmental disabilities

## Abstract

Children with developmental disabilities have different feeding and swallowing problems. The purposes of the present study were to develop an Arabic version of the FHI-C and to evaluate its validity, consistency, and reliability in Arabic children with developmental disabilities for assessing how feeding and swallowing problems impair the physical, functional, and emotional aspects of children’s lives. A prospective study including 113 children [62 children with autism spectrum disorder (ASD), 24 with cerebral palsy (CP), 27 with intellectual disability (ID)], in the age range of 2 to 10 years, selected randomly from the swallowing clinic, phoniatrics unit, Otorhinolaryngology department, University hospital between September 2023 and December 2023 complaining of feeding and swallowing problems. Validity was established by comparing patients` scores to typically developed controls (31 children). For test–retest reliability, forty parents filled out the A-FHI-C again two weeks after their initial visit. Cronbach’s alpha for A-FHI-C was 0.986, indicating good internal consistency. Intraclass correlation showed 0.850 with a 95% confidence interval from 0.779 to 0.898. All three clinical groups had significantly higher total FHI-C and FHI-C domain scores than the control group, indicating good validation. A-FHI-C was found to have significantly high test–retest reliability. The current study indicates that in children with ASD, CP, ID, feeding problems are more prevalent than children who are typically developed. The scores obtained can be used by phoniatricans to evaluate feeding problems and monitor the progress of the therapy plan in children with developmental disorders.

## Introduction

Research indicates that about 1% of the pediatric population experienced swallowing difficulty (3rd Congress of European Society for Swallowing Disorders, 2014; Bhattacharyya, 2015). However, this prevalence is significantly higher in children suffering from severe brain injury, cerebral palsy (CP), children with intellectual disabilities (ID), and autism spectrum disorders (ASD) (Benfer et al., 2012, 2013). Up to 50% of parents in the general pediatric population report having aberrant feeding and swallowing behaviors, and these behaviors subsequently have a negative influence on their children (McGinnis et al., 2019; Pavithran et al., 2019), including malnourishment, dehydration, pulmonary consequences, and even mortality.

Assessment methods for pediatric swallowing problems include both instrumental and non-instrumental approaches (Heckathorn et al., 2016). Although modified barium swallow (MBS) (known as video fluoroscopy) and fiberoptic endoscopic evaluation of swallowing (FEES) are the two most widely utilized instrumental assessment approaches for pediatric dysphagia, the need for self-assessment non-instrument tools is essential (ASHA, 2019).

Non-instrumental assessment tools for pediatric dysphagia focus on the quality of life (QOL) and provide information on how parents or caregivers perceive and rate the impact, function, satisfaction, and prognosis of swallowing problems in their children. On the other hand, instrumental approaches have focused on anatomical, physiological, and pathophysiological measures. However, due to time, expense, the requirement for special equipment, and experienced specialists, it is not easy or practical to carry out these instrumental assessments on all pediatric patients (Vieira & Antunes, 2017).

The available questionnaires that evaluate feeding problems in the general population of children are few and more limited for those with developmental disabilities compared to the tools available for the adult population (Swapna & Anne Maria, 2019). Among the most commonly developed questionnaires designed for the pediatrics population with developmental disabilities are About Your Child’s Eating (AYCE))-revised (Vannatta et al., 2007), Brief Autism Mealtime Behaviour Inventory (BAMBI) (Lukens et al., 2008), Mealtime Behavior Questionnaire (MBQ) (Berlin et al., 2010), Montreal Children’s Hospital (MCH) Feeding Scale (Ramsay et al., 2011) and Feeding Handicap Index for Children (FHI-C) (Shabnam & Swapna, 2023) (Table 1).

**Table 1:** Examples of Arabic validated tools available for feeding and swallowing assessment in children

Test	Authors	Age range	Description
Arabic version of pediatric eating assessment tool (Pedi-EAT-10_Arabic_)	Adel et al. (2022)	6 m–7 y	Pedi-EAT-10_Arabic_ is a valid and reliable parent-report instrument developed to assess symptoms of feeding problems in children and had a strong correlation with FEES for assessment the risk of dysphagia in pediatric population
Arabic version of the child feeding questionnaire (CFQ-A)	Mosli (2020)	2–11 y	CFQ-A is a valid tool to assess parental beliefs, attitudes and practices relating to child feeding. The questionnaire consists of thirty-one items with 5-point Likert response scales
Arabic version of the comprehensive feeding practice questionnaire (CFPQ)	Al-Qerem et al. (2017)	6–12 years	CFPQ is composed of 49 items to explore information about childhood obesity, and factors that potentially influence children’s feeding behavior

Swapna and Shabnam (2023) created FHI for Children (FHIC) to evaluate the feeding problems and their effects on several spheres of life in children with developmental disabilities between the ages of 2 and 10 years old. It is a parent assessment questionnaire of how they perceive their children`s handicapping. It consists of 38 items. It has 21, 12, and 5 items in the three following domains—physical, functional, and emotional—respectively.

All the previously mentioned scales have been developed and standardized on western population. There is a lack of such validated tools in Arabic language, especially to assess the functional, physical, and emotional problems that accompany feeding and swallowing difficulties in children with CP, ASD, and ID.

Currently, there is no available Arabic version of FHI-C. In clinical setting, an Arabic version of the FHI-C could greatly contribute to assessing and monitoring the Arabic-speaking children with swallowing problems. The purpose of the current study is to develop an Arabic version of Feeding handicap index questionnaire, and to evaluate its validity and reliability. Furthermore, the study used the Arabic version of the Feeding Handicap Index (FHI) to evaluate feeding and swallowing issues in the physical, functional, and emotional domains in children with developmental disabilities (cerebral palsy, autism spectrum disorder, intellectual disability including down syndrome) and comparing the results with typically developing children and with other dysphagia assessment tools such as Ped Eat-10 (Arabic version) (Adel et al., 2022).

## Methods

The study received approval from main University’s Institutional Review Board (n.: FMBSUREC/11022024/Lotfy). Prior to testing, the parents of the participants provided written consent after being informed of the study’s objectives. Data collection was done by collaboration of swallowing clinic, Phoniatric unit, University hospital and Special needs center of faculty of postgraduate childhood studies of main University from September 2023 to December 2023.

### Development of A-FHI-C

The Arabic version of FHI-C was translated from the original English version by two Arabic bilingual, skilled phoniatricians (consultants of voice, swallowing, and communication disorders). All questions were then backtranslated into English and compared with the original questionnaire by a certified professional translator familiar with both languages, English and Arabic. The translator then sent the back-translation to the examiners for evaluation and feedback.

### Pilot Study

Twenty parents of children with developmental disabilities who had a history of feeding issues participated in a pilot study in which the A-FHI-C was administered. We observed difficulties in clearly understanding questions 37 and 38 in the Emotional subscale in the original items. So, more explanation words were added for those questions. The participants were given the final version after it had been verified.

### Participants and Procedure

Inclusion criteria: children between the ages of 2 and10 with settled diagnosis of cerebral palsy, intellectual developmental disability, and autism spectrum disorder (ASD). All of them had delayed language and speech and feeding problems. All participants were diagnosed by a skilled multidisciplinary team of medical specialists, comprising pediatricians, psychiatrists, phoniatricians, and psychiatrists, and a psychologist. All participants were referred to feeding clinic at the phoniatric clinic. All the participants had undergone speech—language and physiotherapy rehabilitation.

#### The ASD Group

The ASD group was composed of 50 males and 12 females who had a clinical diagnosis of ASD. They were diagnosed according to the criteria of the Diagnostic and Statistical Manual of Mental Disorders 5th Edition (DSM-V) (American Psychiatric Association, 2013). The severity rating was determined by the Childhood Autism Rating Scale (CARS) (Arabic version) (El-Defrawi et al., 1998).

#### Cerebral Palsy (CP) Group

According to the DSM-5, CP is one of neurodevelopmental disorders. CP group had different severity of intellectual disability (ID) and topographic distributions. There were eight females and sixteen males in the CP group.

#### ID Group

The ID group consisted of three females and twenty-four males who had received a clinical diagnosis based on DSM-V criteria (American Psychiatric Association, 2013). They were selected without any concomitant conditions. According to the nonverbal domain of the Stanford Binet-5th, ID is defined as being two standard deviations or more below the population, or an IQ score of roughly 70 or below. There are four subtypes of ID: profound, severe, moderate, and mild (Abu El-Neel et al., 2011). This group included children with Down syndrome.

#### The TD (Control) Group

The TD group (17 males and 14 females) was selected from preschool nurseries, and schools. They were matched to the ASD, CP, and ID groups for age and gender. Parents of TD children had: (a) no parent concern about child motoric development, no previous complaints of neurological, oro-motor, language or speech disorders, no previous speech therapy, no visual or hearing impairment and language assessment using the modified Preschool Language Scale- Arabic version (PLS-4) was adequate (El Sady et al., 2011).

#### Procedure

Arabic Feeding Handicap Index (FHI) is a 38-item parent assessment tool. It consists of three domains (physical, functional, and emotional domains) with 21, 12 and 5 items in each domain respectively with 3-point rating scale (0, 1, 2) where “0 represents Never has this problem”, “1 represents Sometimes has this problem‟ and “2 represents Always has this problem. At the end of the questionnaire, there is a question about the overall subjective impression of general severity of your child`s feeding problem severity. The response options range from 1 (normal) to 7 (severe difficulty).

Possible scores range of physical domain is (0–42), functional domain is (0–24), emotional domain is (0–10), and total range is (0–76).

All participants completed one study visit during which demographics information was collected, and the parents of all participants filled the A-FHI-C questionnaire after a brief explanation for the questions and possible answers. The time taken to fill the questionnaire was about 30 min.

#### Validation and Statistical Analysis

Validity: We investigated the scale structure using factor analysis. We assessed the Kaiser–Meyer–Olkin (KMO) value and the Bartlett spherical test to evaluate the sampling efficiency and the significant correlation of the data. KMO > 0.60 is the minimum requirement. The appropriateness of the factor analysis was indicated by an important Bartlett test P value. Factor analysis was done according to the Kaiser criterion and factors with a property value of > 1 were considered acceptable. The exploratory factor analysis was performed using PCA with the rotation of Varimax. Spearman correlation rank is used to evaluate the convergence validity of scales.

Reliability: To evaluate internal consistency, we computed the scale’s subscores as well as the overall Cronbach alpha of the total score. Two weeks later, we followed up with a subsample of 40 randomly chosen children to evaluate test–retest reliability. We did this by computing the intra-class correlation coefficient (ICC) for the 38 items, the scores on the individual subscales, and the A-FHI-C total score. A higher correlation coefficient suggests a more reliable test, while a coefficient of ≥ 0.75 indicates excellent agreement.

The Statistical Package for the Social Sciences (SPSS) ver. 26 was used (SPSS, Inc., Chicago, IL, USA). The Mann–Whitney U test was used to compare case and control groups. The results were considered significant when the P value was less than 0.05, and non-significant when it was greater than 0.05.

The power of sample size was estimated using g*power software based on the effect size of 0.5, overall type I error rate (α) ≤ 0.05. One hundred forty-four subjects (113-case, 31-control) are expected to obtain a power of more than 80%.

## Results

This prospective study included 144 children: 31 typically developed (control), 62 ASD, 24 CP and 27 ID with a mean age of 6.24 ± 2.17 years. Detailed clinical characteristics of the studied participants are illustrated in Table 2.

**Table 2:** Descriptive analysis among study participants

No = 144		
Age (mean ± SD)	6.24 ± 2.17	
Gender	Male	107 (74.3%)
	Females	37 (25.7%)
	Frequency	%
Diagnosis		
Control	31	21.53
ASD	62	43.1
CP	24	16.7
ID	27	18.75
Sib Order		
1	46	31.9
2	43	29.9
3	38	26.4
4	16	11.1
5	1	0.7
Mother education		
Illetrate	14	9.7
Prep	3	2.1
Diploma	64	44.4
University	63	43.8
Father education		
Illetrate	16	11.1
Prep	3	2.1
Diploma	62	43.1
University	63	43.8
Family history of mental illness		
No	120	83.3
Yes	24	16.7
Family history of swallowing problem		
No	136	94.4
Yes	8	5.6

For convergent validity, there was a statistically significant strong positive correlation between the total scores of A-FHI-C scale and Eat-10 scale (Table 3).

**Table 3:** Convergent validity analysis: correlation between total score of the FHI scale and the total score of the Eat 10 scale

		Total FHI	Total eat-10
Total FHI	Spearman correlation	1	0.856^**^
	Sig. (2-tailed)	–	0.000
	N	144	144

^**^Correlation is significant at the 0.01 level (2-tailed)

The correlation between the total A-FHI-C value and the patient group’s A-FHI-C subscale value was tested using the Spearman rank correlation coefficient, showing a moderate-high correlation between the total (r = 0 value) and all A-FHI-C subscale values (physical r = 0 value; functional r = 0 value; emotional r = 0 value). The Cronbach coefficient of the total FHI score was 0.947, indicating good internal consistency (Table 4).

**Table 4:** Reliability estimates for the A-FHI scale among all study participants

	Cronbach’s Alpha 0.947
Item	Cronbach’s Alpha if item deleted
FHI1	0.946
FHI2	0.945
FHI3	0.945
FHI4	0.944
FHI5	0.945
FHI6	0.945
FHI7	0.945
FHI8	0.945
FHI9	0.945
FHI10	0.945
FHI11	0.945
FHI12	0.945
FHI13	0.945
FHI14	0.947
FHI15	0.946
FHI16	0.946
FHI17	0.945
FHI18	0.947
FHI19	0.945
FHI20	0.945
FHI21	0.946
FHI22	0.946
FHI23	0.947
FHI24	0.946
FHI25	0.949
FHI26	0.946
FHI27	0.945
FHI28	0.945
FHI29	0.945
FHI30	0.946
FHI31	0.947
FHI32	0.947
FHI33	0.947
FHI34	0.948
FHI35	0.947
FHI36	0.949
FHI37	0.947
FHI38	0.948

For the test–retest reliability, the average measure ICC value was 0.850 with a 95% confidence interval from 0.779 to 0.898 [F test (103,103) = 66.66, *P* < 0.001] (Table 5).

**Table 5:** Intraclass correlation coefficient of the total score FHI (test re-test reliability)

Intraclass correlation coefficient							
	Intraclass correlation	95% confidence interval		F Test with true value 0			
		Lower bound	Upper bound	Value	df1	df2	Sig
Single measures	0.739^a^	0.638	0.815	6.668	103	103	0.000
Average measures	0.850^c^	0.779	0.898	6.668	103	103	0.000

Two-way mixed effects model where people effects are random and measures effects are fixed

^a^The estimator is the same, whether the interaction effect is present or not

^b^Type C intraclass correlation coefficients using a consistency definition. The between-measure variance is excluded from the denominator variance

^c^This estimate is computed assuming the interaction effect is absent, because it is not estimable otherwise

### Comparison of Clinical Groups with the TD (Control) Group

The groups of children with ASD, CP, and ID were compared with the control group for the total A-FHI-C scores and FHI-C domain (Physical, Functional & Emotional) scores. The median and interquartile range (IQR) obtained have been shown in Table 6.

**Table 6:** Median and interquartile range of the studied groups regarding the FHI scale domains

	Diagnosis							
	Control		ASD		CP		ID	
	Median	IQR	Median	IQR	Median	IQR	Median	IQR
Physical	0.00	0.00	5.00	5.5	23.00	21.75	6.00	13.75
Functional	0.00	0.25	6.00	5.0	9.50	11.75	4.00	5.00
Emotional	0.00	0.50	3.00	4.0	2.00	1.75	1.00	2.00
Total	0.00	1.75	14.00	10.25	35.00	30.5	11.50	21.25

### Comparison Between TD Group and the Three Clinical (ASD, CP, ID) Groups for Each Item of the FHI-C

For the ASD group, the Mann–Whitney test results revealed that there was a significant difference between the ASD group and the TD group on the following items 5, 6, 7, 15, 16, 17, 22, 23, 25, 28, 29, 32, 33, 34, 35, 37, and 38. This means that 17 out of 38 items for ASD group (Table 7).

**Table 7:** Comparison between each of the three clinical groups and the control group for each item of the A-FHI-C, A-FHI-C domains, and total A-FHI-C scores (Mann–Whitney test)

	ASD	CP	ID
P1	45.75	34.50*	30.41
P2	46.72	37.46*	32.57
P3	48.97	35.69*	32.96
P4	48.91	39.31*	34.28
P5	52.39*	40.83*	34.24*
P6	50.80*	39.21*	37.33*
P7	50.61	39.67*	33.13
P8	48.59	36.00*	31.30
P9	51.14	38.19*	30.85
P10	48.12	36.31*	31.33
P11	47.11	34.40*	34.35
P12	48.01	35.29*	32.56
P13	47.00	37.88*	35.06*
P14	49.60	38.25*	33.69
P15	53.32*	36.08*	34.31*
P16	51.96*	35.50*	35.07*
P17	54.85*	36.38*	31.98
P18	46.48	33.29*	31.35
P19	48.02	36.67*	33.07*
P20	47.52	34.71*	32.52*
P21	47.26	32.06	33.61*
F22	53.81*	33.75*	34.39*
F23	54.60	34.23*	33.20*
F24	51.34	37.54*	36.15*
F25	58.43*	35.17*	33.78*
F26	48.45	37.25*	31.31
F27	49.30	35.04*	32.70*
F28	52.50*	37.04*	33.52*
F29	56.04*	37.48*	35.89*
F30	49.14	34.52*	33.17*
F31	47.02	32.10	33.07*
F32	54.25*	32.52*	31.22
F33	53.65*	32.94*	30.26
E34	54.40*	33.90*	31.11
E35	52.49*	32.88	31.20
E36	51.69	34.29*	34.43*
E37	52.25*	31.23	31.80*
E38	54.06*	30.13	33.06*
Physical	56.06*	40.67*	37.00*
Functional	60.10*	39.79*	38.06*
Emotional	57.49*	38.27*	36.59*
Total FHI	58.50*	40.88*	38.80*

^*^P ≤ 0.05

Comparably, the results of the Mann–Whitney test of the CP group showed that, with the exception of items 21, 31, 35, 37, and 38, there was a significant difference between the two groups on each item. This implies that 33 out of 38 items, or the majority, were impacted in children with CP, indicating the severity of feeding problems in these children (Table 7).

ID group reported that there was a significant difference on all items except on items 1, 2, 3, 4, 7, 8, 9, 10, 11, 12, 14, 17, 18, 26, 32, 33, 34 and 35 between both the groups. This indicated that 20 out of 38 items for ID group (Table 7).

Physical, functional, emotional, and total FHI are significantly affected in the three groups under the study. Table 7 depicts the Mann–Whitney values obtained for each item for all the three clinical groups.

## Discussion

Quality-of-life assessment tools for children who experienced feeding and swallowing problems are very crucial to report and monitor their impact of swallowing dysfunction on various domains of life and assess how much does it limit their ability to participate fully in society. The current study investigated the feeding and swallowing problems in children with developmental disabilities (ASD, CP, ID including down syndrome) using A-FHI-C.

The A-FHI-C was administered to 113 children with DD and 31 TD children. The results of this study suggest that the A-FHI-C has good internal consistency with a Cronbach’s α coefficient value of 0.947. This is consistent with the results of the original version of FHI-C (Shabnam et al., 2023). The Cronbach’s α value of 0.95 reported in the original FHI-C. Furthermore, the A-FHI-C items showed a significant correlation within item-to-item and item-to-total score (r ranging from 0.944 to 0.949).

Test–retest reliability is evidenced by high values of both intraclass correlation and Spearman’s rank coefficient (0.739 and 0.856, respectively). The reliability of A-FHI-C instrument is confirmed by the good internal consistency of the A-EAT-10 items and the highly significant correlation found between its items and its total score.

Given the intraclass correlation and Spearman’s rank coefficient values (0.739 and 0.856, respectively), test–retest reliability also suggested high scores. The test–retest results exhibit a strong correlation, indicating the high reproducibility and stability of the A-EAT-10’s psychometric properties. The strong internal consistency of the A-EAT-10 items and the highly significant correlation observed between its items and total score confirm the validity of the A-FHI-C instrument.

As compared to control group, children with ASD, CP, and ID exhibited more severe feeding problems. These results agree with previous literature (Badalyan & Schwartz, 2011; Bandini et al., 2010; Gangil et al., 2001; Marshall et al., 2014; Nadon et al., 2011; Rajshree & Manjula, 1991; Stallings et al., 1996; Trier & Thomas, 1998).

### ASD and Feeding Problems

ASD is a type of neurodevelopmental disorder (NDD) that is marked by social communication and social interaction deficits, as well as restricted stereotyped repetitive patterns of behavior, interests, and activities (American Psychiatric Association, 2013).

The current study entailed that, children with ASD had several feeding problems such as difficulty using spoon independently, or for scooping food, inappropriate weight gain. They keep the food in the mouth for a long time without swallowing, refuse to eat, difficulty swallow solid and semi-solid food, avoid solid food, eat less, and refuse newly introduced food based on the taste/temperature/texture/smell. Mothers are forced to push the food to the child’s mouth, pinch the child’s nose, shake child’s head/face, or even close the child lips/jaw to make him swallow the food. Children with ASD frequently have prolonged mealtime, exhibit frustration and temper during feeding, especially in social gatherings.

The primary feeding characteristics in children with ASD include food refusal, food selectivity (based on particular food shapes, colors, textures, presentation, or arrangements on the plate), and disruptive mealtime behaviors (Cermak et al., 2010; Schreck et al., 2004; Shabnam et al., 2019).There is a tendency to limited range of food preference especially carbohydrates, food neophobia, requiring assistance during eating, eating non-food items instead of food, ritualistic feeding behaviors, poor self-feeding skills, and mealtime behavior problems. Mothers are compelled to use different strategies to ensure that their children complete their meals (Mahmoud et al., 2021).

### CP and Feeding Problems

According to parent responses, children with CP revealed difficulty sucking from bottle or breast, difficulty biting and chewing hard food (biscuit) and/or soft food (cake), difficulty eating independently with fingers or with spoon, difficulty using the spoon for food scooping, difficulty clearing the food from the spoon with the lip, difficulty drinking independently or through a straw or cup, drooling while feeding, difficulty swallowing different food consistencies (liquid, semisolid & solid), difficulty clearing the stuck food in mouth, difficulty rinsing after eating, inappropriate weight gain, retain the food in mouth for a long time without swallowing, having prolonged meals, and not to open mouth while feeding. In addition, they have nasal regurgitation, gaging, vomiting, eat less, avoid giving solid food, refusing newly food based on the taste/temperature/texture/smell. Parents may need to place their children in a specific position or chair during feeding, push food or milk back, pinch child’s nose, shake child’s head to ensure that the food is swallowed. They feel tempered during mealtime while feelings of embarrassment or upset during social situations, or being dependent on others were not reported in this study. These findings were in consistent with the original study and other studies (Arvedson, 2013; Diwan & Diwan, 2013; Gangil et al., 2001; Ghayas et al., 2014).

### ID and Feeding Problems

According to our findings, children with ID have challenge to use spoon for scooping food or pick the food by their lips, difficulty clearing the stuck food in the mouth with tongue, inappropriate weight gain, taking longer to complete a meal, and retention of food in the mouth without swallowing for a long time. A child eats less, coughs, spills, gags, vomits, and chokes when giving solid or liquid food, avoids solid food, avoids new food because of their taste, temperature, texture, or smell, and requires smaller meals. Mothers push the food or milk into the child`s mouth to ensure that the food is swallowed. Special feeding products (feeding tube, special feeding bottles) and utensils (such as a spoon, plate, etc.) are frequently required for children with ID. They usually feel upset about not eating independently or sharing social meals.

These findings are once again in with the results of previous studies, show that the most prevalent feeding problems are inability to feed independently, the need for special equipment, and the need for special positioning during feeding. The delay in motor development may also result in further motor limitations (Lahtinen et al., 2007; Matson et al., 2008; Rezaei et al., 2011; Shabnam et al., 2019).

### TD (Control) Group and Feeding Problems

For TD children, the majority of the answers were “never”, indicating that there were no serious feeding issues in this group. However, few parents responded ‘Always’ to some questions.

6.5% of parents of TD children stated problems with items 2, 8, 9, 10, 11, 24, 30 which difficulty biting hard and/or soft food, drinking liquid from a glass/cup, drinking independently through a straw, drooling, and spilling the solid or liquid food during feeding. 6.5% of parents have to pour milk into the child`s mouth to make certain that the food is swallowed. 9.7% of parents reported feeding problems including using spoon independently, holding the solid/ liquid food in mouth, and being dependent on others for feeding. Approximately 13% of the parents asserted that their children had difficulty in rinsing and spitting water after eating. However, they reported that their children weren’t trained on rinsing water during washing their mouths.

### Study Limitations

As feeding problems in children with DD are prevalent in early life neonate, toddlers, children and may continue in later adolescent life. Early assessment and follow up with multidisciplinary approach are crucial to avoid risk of feeding problems such as malnutrition, respiratory problems, and even aspiration. Future studies should include the validation of the Arabic version of the tool on adolescents.

As other studies using questionnaire, we should have agreement with instrumental methods such as videofluroscopy or fiberoptic endoscopic evaluation of swallowing (FEES).

Different variables should be investigated for correlation with feeding problem in future research such as time of onset of the feeding related complaints, severity of the problem, associated comorbidities such as language disorders, speech disorders, social impairment, …. in the three groups under the study. Limited studies investigate ASD severity whether related to feeding problems or not. Some studies suggest that feeding problems are related to ASD severity (Schreck et al., 2004) while others refute this claim (Crasta et al., 2014; Mahmoud et al., 2021).

## Conclusion

This study provides a new validated and reliable self-assessment tool for assessing the physical, functional, and emotional aspects related to feeding in children with developmental disabilities. Compared to the TD group, all three clinical groups (ASD, CP, and ID) had significantly higher total A-FHI-C scores as well as scores for each domain. This suggested that the developed tool had good clinical validity. High alpha values were indicative of test–retest reliability. Therefore, phoniatricans can assess feeding problems and monitor the progress of the therapy plan in children with developmental disabilities.

## Data Availability

Available from corresponding author upon request.
